# Gender, Age and Season as Modifiers of the Effects of Diurnal Temperature Range on Emergency Room Admissions for Cause-Specific Cardiovascular Disease among the Elderly in Beijing

**DOI:** 10.3390/ijerph13050447

**Published:** 2016-04-27

**Authors:** Shan Zheng, Minzhen Wang, Bei Li, Shigong Wang, Shilin He, Ling Yin, Kezheng Shang, Tanshi Li

**Affiliations:** 1 Institute of Epidemiology and Statistics, School of Public Health, Lanzhou University, Lanzhou 73000, China; wangmzh@lzu.edu.cn; 2 General Hospital of PLA, Beijng 100853, China; libei301@163.com (B.L.); heshilin301@163.com (S.H.); yinling301@126.com (L.Y.); 3 College of Atmospheric Sciences, Lanzhou University, Lanzhou 730000, China; wangsg@lzu.edu.cn (S.W.); shangkz@lzu.edu.cn (K.S.); 4 School of Atmospheric Sciences, Chengdu University of Information Technology, Chengdu 610225, China

**Keywords:** diurnal temperature range, modifiers, elderly, emergency room admission, cardiovascular disease, hypertensive disease, ischemic heart disease, cerebrovascular disease

## Abstract

*Background*: Diurnal temperature range (DTR) is an important index of climate change and variability. It is also a risk factor affecting human health. However, limited evidence is available to illustrate the effect of DTR modification on cause-specific cardiovascular disease among the elderly. *Methods*: A semi-parametric generalized additive model (GAM) was used to analyze the exposure-effect relationship between DTR and daily emergency room (ER) admissions for cause-specific cardiovascular diseases among the elderly from 2009 to 2011 in Beijing. We examined the effects of DTR for stratified groups by gender and age, and examined the effects of DTR in the warm season and cold season for cause-specific cardiovascular diseases. *Results*: Significant associations were found between DTR and ER admissions for all cardiovascular and cerebrovascular disease among elderly males, while DTR was significantly associated with ER admissions for all cardiovascular disease, ischemic heart disease and cerebrovascular disease among elderly females. People aged 75 years and older were more vulnerable to DTR. DTR caused greater adverse effects on both genders in the warm season, whereas the effect estimates on females were higher in cold season than in warm season. *Conclusions*: A short-term increase of DTR was significantly associated with ER admissions for cause-specific cardiovascular disease among the elderly in Beijing. Gender, age and season may modify the acute health effect of DTR. Some prevention programs that target the high risk subgroups in the elderly for impending large temperature changes may reduce the impact of DTR on people’s health.

## 1. Introduction

In recent years, more and more attention has been paid to the association between climate change and human health. It has been reported that the ambient temperature, the key climatic change indicator, is able to lead to not only direct deaths and illnesses, but also the aggravation of cardiovascular and respiratory diseases [1,2,3,4]. Besides that, diurnal temperature range (DTR), defined as the difference between maximal and minimal temperature within one day, is considered as an important index of climate change and variability because it provides more information than the mean temperature alone [5]. Due to the fact that during the 20th century, the minimum temperature raised three times quicker than the maximum temperature, DTR is currently decreasing in most parts of the world [6,7]. The reason for the observed changes in DTR might be related to both large-scale climate forcing and local effects [7,8]. Although DTR is decreasing in the context of long-term climate change, important short-term impacts of large DTR on human health are continuously found in many countries and regions, which still supports the notion of DTR being a risk factor for human health.

Until now, the adverse effects of higher DTR have been observed to be associated with mortality and morbidity. Most of these studies targeted the association between DTR and human death, including non-accidental, cardiovascular, and respiratory mortality, and significant effects were shown in Hong Kong [9], Taiwan [10], mainland China [11], Japan [10], Korea [12] and Canada [13]. Only a small portion of studies based on emergency and hospital data showed significant effects of DTR on cardiovascular and respiratory morbidity [14,15,16,17]. These studies mostly focused on the relationship between DTR and broad categories of cardiovascular and respiratory causes of death and morbidity. Information about the association between DTR and subgroups of cardiovascular disease of emergency room (ER) admissions is quite scarce. The elderly, who are more sensitive to temperature variations, constitute the largest proportion of cardiovascular deaths. Meanwhile, it has been reported that they were also a susceptible population affected by DTR [10,18]. Nevertheless, few studies have focused on the association between DTR and cause-specific cardiovascular disease among the elderly. In addition, less evidence is available to illustrate the effect modification of DTR, especially in cities of developing countries with a high risk of DTR exposure. Hence, there is a need to explore the adverse effects of DTR on cause-specific cardiovascular diseases morbidity, mortality and these modifying effects among the elderly.

In the present study, a time-series analysis was conducted to examine the modifying effect of gender, age and season on the association between DTR and ER admissions for cause-specific cardiovascular diseases among the elderly in Beijing, China. Better understanding of the effect modification of DTR on morbidity among the elderly, will provide useful information for the development of public health plans and risk assessments in the ambient environment.

## 2. Materials and Methods

### 2.1. Emergency Room Admissions Data

Data on daily ER admissions were collected from 1 January 2009 to 31 December 2011 from three large-scale comprehensive hospitals in the Haidian District of Beijing. The cases concerning people aged 65 years or older were extracted. The causes of ER admissions were classified according to the International Classification of Disease, tenth revision (ICD-10) into all cardiovascular disease (ICD10: I00~I99, G45~G46), hypertensive disease (ICD10: I10~I15), ischemic heart disease (ICD10: I20~I25) and cerebrovascular disease (ICD10: I60~I69, G45~G46). ER admissions data included admission date, age, gender, residential address, primary diagnosis and diagnostic code. Primary diagnoses data were used in this study. This study was approved by the medical ethics committee of school of public health of Lanzhou University (No. 2015-01).

### 2.2. Meteorological and Air Pollution Data

Daily meteorological data during the study period, including daily minimum, maximum and mean temperature (T), relative humidity (RH), mean wind speed (W) and atmospheric pressure (P), were obtained from the Beijing Meteorological Bureau. The weather data were measured at a fixed-site station located in the Daxing District of Beijing. DTR was calculated as the difference between the maximum and minimum temperature within one day. Due to the possible confounding caused by the association between air pollution and morbidity [19], we also accessed daily data on urban sulfur dioxide (SO_2_), nitrogen dioxide (NO_2_), and particulate matter less than 10 μm in aerodynamic diameter (PM_10_) from the Beijing Public Net for Environmental Protection website [20].

### 2.3. Statistical Methods

The Poisson generalized additive model (GAM) was used to analyze the associations between DTR and daily ER admissions of the elderly. Firstly, we built an independent model to explore the patterns of the relationships between DTR and the ER admissions. The regression splines function (s) was used to control for long-term trends and seasonal patterns, as well as the weather variables [21]. The partial autocorrelation function (PACF) was used to select the degree of freedom of time trends until the absolute values of the sum of PACF for lags up to 30 reached a minimum. Specifically, the degrees of freedom (df) per year for time trends were defined for both genders in all cardiovascular disease (df = 4, 5), hypertensive disease (df = 4, 5), ischemic heart disease (df = 4, 5) and cerebrovascular disease (df = 5, 5). For weather conditions, 3 df was used for mean temperature, relative humidity, mean wind speed and atmospheric pressure during the whole study period [21,22]. Additionally, we adjusted for the day of the week (DOW) and public holidays (Holiday) as dummy variables in the model. Residuals of the basic models were used to check whether there were discernable patterns and autocorrelation by means of residual plots and PACF plots [23]. In order to control for potential confounding effect, when the absolute magnitude of the PACF plot was large than 0.1 for the first two lag days, the core models were added a regressive term of ER admissions on lag1 day to remove the autocorrelation of residuals [24,25]. Also, the pollutants (PM_10_, NO_2_ and SO_2_) were included as confounders in the model. Secondly, a categorical variable with two categories was created to denote the season level. The whole data were divided into two periods: warm season (from May to October) and cold season (from November to April). A binary variable in each season was created, with 1 for the selected season and 0 for other season. Then to test the possible interaction between DTR and the two seasons of days, a product term of DTR and the season level was added as dummy variables into the core model. The varied DTR effects across two seasons were estimated to explore the possible modification of the season on the effect of DTR [14,24].

In addition, we examined associations stratified by gender (female and male) and age (65–74 years, ≥75 years) for cause-specific cardiovascular disease. The statistical significance of differences between effect estimates for subgroups (e.g., female VS male, 65–74 years VS ≥ 75 years) was examined by calculating $\left(\beta_{1}-\beta_{2}\right)/\sqrt{SE_{1}{}^{2}+SE_{2}{}^{2}}\;$, where β_1_ and β_2_ were the estimates for the two categories, and SE_1_ and SE_2_ were their respective standard errors [26,27]. An absolute value larger than 1.96 was considered as a statistically significant difference at α = 0.05 level. Regardless of significance, modification of effect by a factor of ≥2 was considered to be important and worthy of attention [22,27]. Delayed effects were considered to investigate with single day lags (from L0 to L4) and cumulative day lags (from L01 to L04) for DTR. The lagged day with the largest estimated effect in the core model was chosen to analyze the other steps in the study. The smoothing function was also used to graphically analyze the significant effect of DTR on ER admissions. We included long-term trend of admissions, DOW, and Holiday compared the effects of DTR in the single-variable models and multiple-variable models with a different combination of weather and air pollution variables. The estimated effects were expressed as the increased percentage and their 95% confidence interval (95% CI) of the daily ER admissions of the elderly with a 1 °C increase in DTR. All analyses were run on the R 3.2.3 statistical software (The R Foundation: Vienna, Austria) using the mgcv package.

## 3. Results

### 3.1. Descriptive Statistics

The daily cause-specific cardiovascular ER admissions among the elderly during 2009–2011 in Beijing are shown in Table 1. From 2009 to 2011 (1098 days), a total of 22,069 elderly ER admissions for all cardiovascular disease (11,049 males and 11,020 females) were recorded in the study. For the other causes of disease, the accounts of hypertensive disease, ischemic heart disease and cerebrovascular disease were 6051, 6735, and 8202, respectively. On average, there were approximately 8.96 and 11.19 ER admissions per day for people aged 65–74 years and ≥75 years, respectively. During the study period, the minimal, mean and maximal DTR were 1.10 °C, 9.85 °C and 21.90 °C. The mean DTR was slightly lower in cold season than in warm season. The mean temperature, relative humidity, mean wind speed and atmospheric pressure were 13.15 °C, 50.38%, 2.23 m/s and 1012.38 hpa, respectively. The average concentrations of PM_10_, SO_2_ and NO_2_ were 110.16 μg/m^3^, 28.07 μg/m^3^ and 51.88 μg/m^3^, respectively (Table 2).

### 3.2. Effects by Gender

Table 3 shows the estimates for the percent increase in elderly ER admissions for cause-specific cardiovascular disease associated with a 1 °C increase in DTR by gender in different lag structures after adjustment for long-term and seasonal trends, weather, air pollution and other confounders. Generally, the cumulative-day metrics of DTR exposure showed a larger effect on elderly ER admissions than single-day exposures except for hypertensive disease ER admissions. The differences between males and females for the effects of DTR on cause-specific cardiovascular disease ER admissions were observed. For all cardiovascular and ischemic heart disease ER admissions, the effects of DTR in females were higher than in males. A 1 °C increase in the 2-day and 3-day moving average of DTR (lag 01 and lag 02) corresponded to a 1.46% (95% CI: 0.74%~2.19%) increase in all cardiovascular disease ER admissions and 1.71% (95% CI: 0.21%~3.23%) increase in ischemic heart disease ER admissions among females. A significant association was found for all cardiovascular disease ER admissions with DTR at lag 03 and 04 day among males only. For cerebrovascular disease ER admissions, the effect of DTR in males with cumulative-day lag was higher than in females. We did not observe a significant effect of DTR on hypertensive disease ER admissions in either gender. No significant difference was found between the two genders in any of the ER admissions for cause-specific cardiovascular disease.

**Figure 1:**
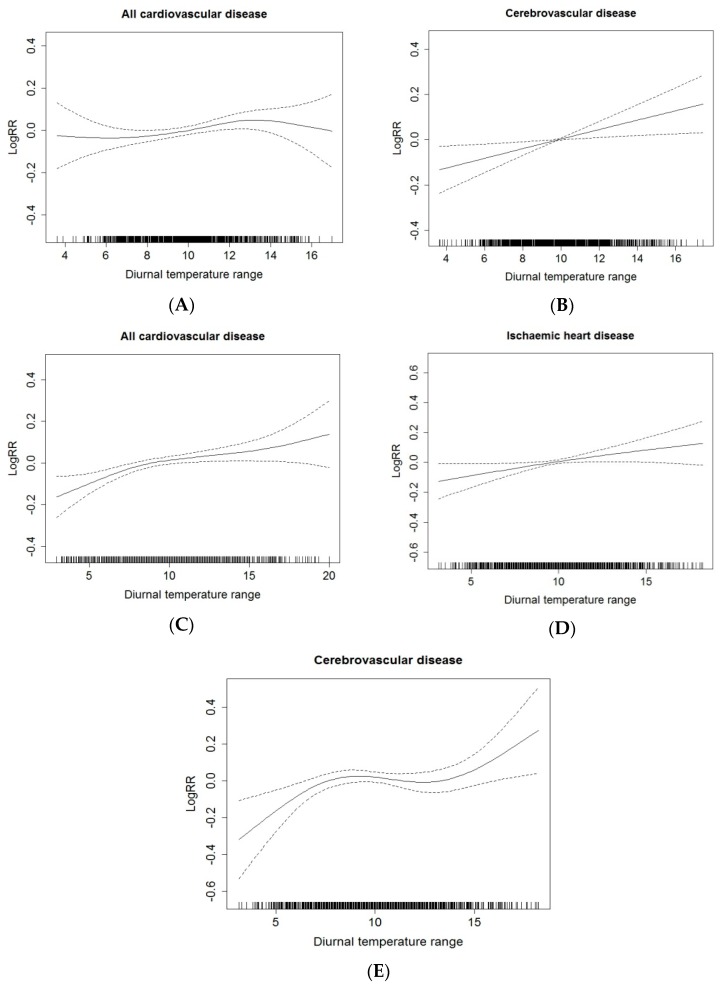
Smoothing plots of DTR against the elderly ER admissions risk of cause-specific cardiovascular disease. X-axis is DTR (°C). The solid lines indicate the estimated mean percentage of change in daily ER admission, and the dotted lines represent twice the standard error. (**A**,**B**) The relationship between DTR and all cardiovascular disease (L04 day), cerebrovascular disease (L04 day) ER admissions among males; (**C**–**E**) The relationship between DTR and all cardiovascular disease (L01 day), ischemic heart disease (L02 day) and cerebrovascular disease (L02 day) ER admissions among females. Models controlled for time trend, DOW, holiday, mean temperature, humidity, wind, atmospheric pressure and air pollutant concentrations.

Figure 1 shows the exposure-response relationships between DTR and the elderly ER admissions for cause-specific cardiovascular disease among both genders at the significantly greatest lagged-day after controlling for confounders in Beijing during study period. There were similar positive linear relationships between DTR and the elderly ER admissions among both genders. It demonstrated that the relative risk of the elderly ER admissions for cause-specific cardiovascular disease increased as DTR increased in Beijing. Since there was no significant association between hypertensive disease ER admission and DTR, the corresponding figure is not shown there.

### 3.3. Effects by Age

**Figure A1:**
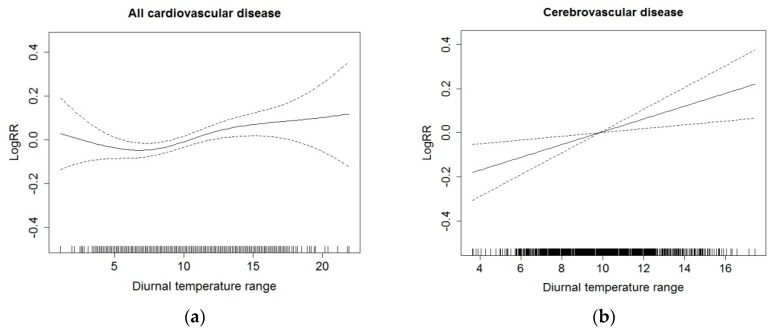

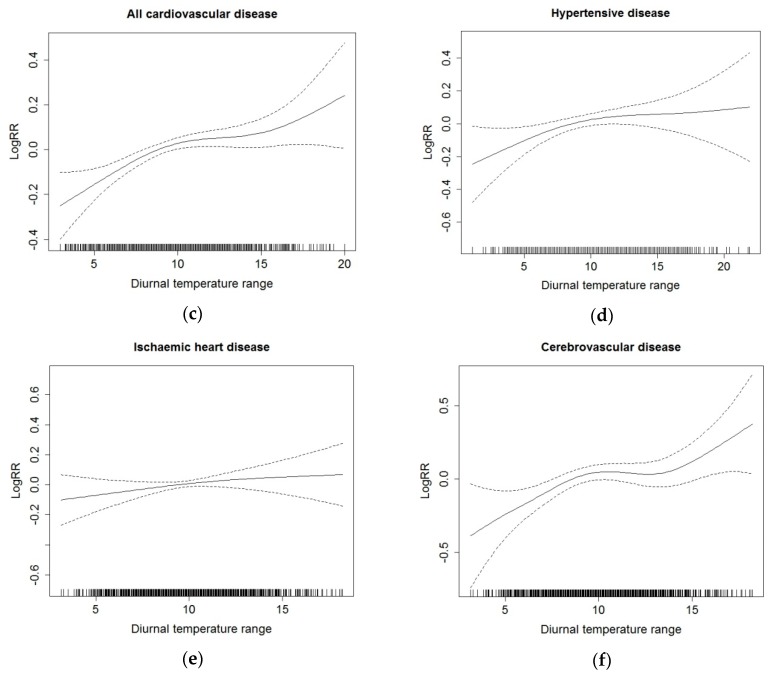
Smoothing plots of DTR against the ER admissions risk of cause-specific cardiovascular disease in the age group 75 years and over. (**a**,**b**) The relationship between DTR and all cardiovascular disease (L04 day) and cerebrovascular disease (L04 day) ER admissions among males aged 75 years and over; (**c**–**f**) the relationship between DTR and all cardiovascular disease (L01 day), hypertensive disease (L0 day), ischemic heart disease (L02 day) and cerebrovascular disease (L02 day) ER admissions among females aged 75 years and over. Models were controlled for time trend, DOW, holiday, mean temperature, humidity, wind, atmospheric pressure and air pollutant concentrations.

Table 4 shows that magnitude of the effects of DTR on the elderly ER admissions for cause-specific cardiovascular disease varied with gender and age. For the elderly aged 65–74 years, the effect estimates of DTR were insignificant in both genders. Among those aged 75 and over, the effect estimates of DTR were significant, with the exception of the elderly male ER admissions for hypertensive disease and ischemic heart disease. The effect estimates of DTR among females aged 75 and over were approximately 1–2 times higher than that among males aged 75 and over, except for the effect estimates of DTR on ischemic heart disease ER admissions in female. Between-age differences among females were significant for all cardiovascular disease and cerebrovascular disease ER admissions. Significant exposure-response relationships between DTR and ER admissions for cause-specific cardiovascular disease among the elderly persons aged 75 years and over were shown in Figure A1, and the positive linear relationships were similar to the effect of DTR on total elderly people.

### 3.4. Effects by Season

Table 5 shows the effects of DTR on the elderly ER admissions for cause-specific cardiovascular disease across the two seasons with the interaction terms of DTR and season. In the warm season, significant positive relationships were observed between DTR and all cardiovascular disease and cerebrovascular disease ER admissions in females. Significant association of DTR with cerebrovascular disease ER admission among males was also observed, with a higher effect estimate than that among females. Among people aged 75 years and over, DTR was significantly associated with all cardiovascular disease and cerebrovascular disease ER admissions, and the effect estimates were higher than among the total elderly in both genders. In cold season, the effect estimates of DTR were significant only in females, whereas the effect estimates of DTR on cerebrovascular disease ER admission was not significant in all age groups. Compared with the total elderly, the effect estimates of DTR were also larger in females aged over 75 years. The season differences only in cerebrovascular disease ER admission were significant among total males and males age 75 years old.

### 3.5. Single and Multiple Models

Table 6 compares the regression results of single-variable and multiple-variable models. The covariates, such as mean temperature, relative humidity, mean wind speed, atmospheric pressure and air pollutants (PM_10_, NO_2_ and SO_2_), were introduced gradually into the multiple-variable models, and only DTR was considered in the single-variable model. The effect estimates of DTR on all cardiovascular and cerebrovascular disease ER admission remained statistically significant before and after adjustment for the covariates in both genders. A similar result was observed for ischemic heart disease ER admission among females.

## 4. Discussion

In this time series study, we examined the relationship between diurnal variations in temperature and the ER admissions for cause-specific cardiovascular disease among the elderly in Beijing. We found significantly acute effects of DTR on all cardiovascular and cerebrovascular disease ER admissions among males. Among females, DTR was significantly associated with ER admissions for all cardiovascular disease, ischemic heart disease and cerebrovascular disease. We observed stronger effects of DTR in people aged 75 years and over. The DTR caused greater adverse effects on both genders in warm season, but the effect estimates on females were higher in cold season than in warm season. The associations between DTR and ER admissions for cause-specific cardiovascular disease were robust to the adjustment for weather conditions and air pollutant concentrations.

Previous studies have shown the significant associations between DTR and cardiovascular mortality and morbidity. A recent study on 30 cities in China, Japan, Korea and Taiwan, China showed that DTR was associated with increased risk of death [10]. In most cities, the adverse health association was greater for circulatory mortality and among the elderly, and the effect estimates of DTR among those ≥65 years varied from 0.45 (−0.76, 1.68) to 1.58 (0.00, 3.18) with cardiovascular mortality. In Korea [16], a multicity study on the risk of hospital admissions for various cardiovascular diseases showed that DTR had adverse effect on cardiac failure with an effect estimates of 3.0 (1.4, 4.6). In Hong Kong (China) [15], the adverse effect of DTR was associated with emergency heart failure admissions with an effect estimate of 0.86% (0.31%, 1.43%), and DTR exhibited a significantly greater effect on elderly patients (aged ≥75 years). In Japan [28], a cohort study showed that all stroke and intracerebral hemorrhage in men aged 40 years and over was significantly related to intradiurnal temperature change. Our current study showed a greater effect of DTR on ER admissions for cause-specific cardiovascular diseases among the elderly, which was consistent with these reported studies. However, the evidence of the relationship between DTR and cardiovascular-specific morbidity was limited, with most of the findings concentrated in Asia. Similar studies needs to be conducted to assess DTR effects in other regions.

In the present study, we found that gender and age may modify the health effects of DTR. Compared with the younger population, the elderly were more susceptible to the adverse impact of DTR, which is consistent with prior studies in China [11,15], Korea [12] and Japan [10]. We also found the effects of DTR on all cardiovascular disease and ischemic heart disease ER admissions were greater in females than in males, and the effect of DTR on cerebrovascular disease ER admissions was reversed in both genders. However, results from prior studies on gender-specific effects of DTR were inconsistent. For example, Yang *et al.* [29] found that women in Guangzhou (China) were consistently more sensitive to the adverse effects of DTR on the total and cardiovascular mortality. Meanwhile, Lim *et al.* [12] found the similar results of deaths among females, which were associated more strongly with DTR than among males in six Korean cities. Qiu *et al.* [15] also reported significant effects of DTR on emergency hospital admissions for heart failure among females in Hong Kong (China). In contrast, Kan *et al.* [8] found significant effects of DTR on total mortality in both genders without a statistical significant difference by gender in Shanghai (China). Lim *et al.* [16] did not observe any gender-based DTR effects on cardiovascular and respiratory hospital admissions in four Korean cities. In previous systematic reviews on the effect modifiers of the temperature-mortality or -morbidity relationship, the differences of the temperature effects by gender were dependent on location and population [1,30], but evidence for modifiers of the DTR-health relationship in previous studies was limited, so the reasons for our gender-specific observations are unclear and deserve further investigation.

We also found that season may modify the effect of DTR. In our study, significant effects of DTR on both genders were observed in warm season, and the adverse effects on females were found to be higher in cold season than in warm season. According to previous studies, the joint effects between DTR and seasons on health were rarely explored and not explicitly indicated [18]. Lim *et al.*’s study [12] showed that DTR was the greatest threat to vulnerable study populations, with greater influence in fall. The greater estimated effects of DTR were also observed in cold season in Hong Kong (China) on emergency heart failure admissions [15] and in eight Chinese cities on total and cardiovascular mortality [11], which implied the interaction between DTR and season. In our previous study, we also found that the significant effects of DTR on four major causes of ER admissions varied in spring, autumn and winter [14]. For the present study, DTR was slightly larger in warm season than in cold season in Beijing, which may explain the fact that more adverse effects of DTR were found in warm season. Kim *et al.* [10] found the greater DTR effect in the higher DTR areas with the results from the meta-analysis in East Asia accordingly. In addition, women have been reported to have a greater risk from temperature change for coronary events and ischemic stroke in cold periods than do men [31,32]. In our study the stronger effect estimates of DTR among females in cold season were more likely to be caused by the cold effect.

Elderly people have been widely regarded as a susceptible population for temperature change [18,33]. They are less able to regulate body temperature, which thus may be more prone to adverse effects of temperature variation. A large change in temperature within one day may cause a sudden change in the heart rate and circulation of elderly people, which all may act to increase the risk of cardiovascular and other diseases, even lead to fatal consequences. Up to now, the physiological mechanism of DTR on cardiovascular disease has not been elucidated, although there are possible explanations on its effects. Temperature-induced damage is thought to be related to enhanced sympathetic reactivity followed by activation of the sympathetic nervous system, renin-angiotensin system, as well as dehydration and a systemic inflammatory response [34]. Several cold pressor tests showed that the hemodynamic stresses and neurohumoral activation in association with temperature reduction were able to exacerbate heart failure, induce myocardial ischemia and precipitate arrhythmias [35,36,37]. Under this background, the elderly with cardiovascular diseases may not adjust well to sudden outside temperature variation due to a decline in immune function and organs. Therefore, studies on the underlying mechanisms, by which temperature variation induces pathophysiological response and cardiovascular diseases, await profound and lasting investigation.

According to the data of the World Health Organization (WHO), cardiovascular diseases were the leading cause of non-communicable diseases (NCD) around the world in 2012, accounting for 46.2% of NCD deaths, or approximately 30% of all global deaths [38]. Most previous epidemiological studies indicated that both low and high temperatures contributed to the increased risks of cardiovascular mortality or morbidity [1,33,39,40]. Two decades of research have also shown that air pollution can trigger heart attacks, strokes, and irregular heart rhythms, particularly in people whom already at risk for these conditions [19,41,42]. Our findings of significant associations among mean temperature, air pollution and cardiovascular disease ER admissions within the elderly, are in consistent with those prior studies (Table A1). However, the effect of DTR on ER admissions for cardiovascular disease remained statistically significant before and after adjustment for mean temperature and air pollutants in multiple models. Our results showed that the change in covariates for weather conditions and air pollutants did not substantially affect the estimated effects of DTR, suggesting that DTR is an independent risk factor for ER admissions among the elderly.

Our findings have potentially important health management implications. First, increased vigilance for large diurnal temperature fluctuations is important in elderly patients with cardiovascular disease, in order to prevent and control clinical symptoms at an early stage. Early warning systems for impending large temperature changes may reduce the impact of DTR on people’s health [9]. Elderly females and people aged 75 years and older with cardiovascular disease should be monitored closely by family members or healthcare workers. They should be given advice on appropriate clothing and timely medication use for sudden temperature changes. Second, age, gender and season may modify the health effects of DTR on ER admissions for cardiovascular diseases in Beijing. Third, the effect of temperature change on morbidity emphasizes the need to better understand the underlying mechanisms.

In addition, our study has several limitations. Firstly, the data of ER admissions were only collected from three hospitals in Beijing, so a selection bias may exist. Secondly, we only used weather condition data from one meteorological station as a surrogate of personal exposure levels to diurnal temperature ranges. The use of ambient rather than personal exposure measurements is expected to result in exposure misclassification. However, the difference between these proxy values and the true exposures is an inherent and unavoidable measurement error. Thirdly, we did not analyze the effect on DTR modification by socioeconomic and educational level in our study. Although significant DTR effects after a modification by socioeconomic and educational level were found in a few studies [12,29], we would not collect that information from emergency room records. Fourth, we did not consider the difference between first-time ER admissions and people with a history of ER admissions, which may affect our results. Finally, since temperature variation may occur both indoors and outdoors, a few studies found that ownership and usage of air conditioning might reduce the effects of temperature on some health outcomes [43,44]. In addition, a large difference between outdoor and indoor temperature may have short-term impact on heart rate and blood pressure [45]. We did not analyze the potentially confounding effect of air conditioning and the effect of indoor-outdoor temperature differences due to the lack of necessary data, which suggests research directions for another future in-depth study.

## 5. Conclusions

We observed short-term associations between DTR and increased ER admissions for cause-specific cardiovascular diseases among the elderly in Beijing during 2009–2011. Furthermore, our results suggest that age, gender and season may modify the acute health effect of DTR on ER admissions. These findings provide new information about the effects of modifiers on the relationship between DTR and morbidity in Beijing and may have implications on local environmental and social policies.

## Figures and Tables

**Table 1:** Distribution of daily ER admissions for cause-specific cardiovascular disease among the elderly in Beijing (2009–2011).

Cause-Specific Cardiovascular	Group	Number	Mean	SD	Min.	P_25_	P_50_	P_75_	Max.
All cardiovascular disease	Total	22069	20.15	6.23	4	16	20	24	43
	Male	11049	10.09	3.66	2	8	10	13	24
	Female	11020	10.06	4.02	0	7	10	13	24
	65–74	9814	8.96	3.53	1	7	9	11	23
	≥75	12255	11.19	4.27	0	8	11	14	26
Hypertensive disease	Total	6051	5.53	2.81	0	4	5	7	14
	Male	2601	2.38	1.67	0	1	2	3	8
	Female	3450	3.15	2.02	0	2	3	4	13
	65–74	2847	2.60	1.81	0	1	2	4	12
Ischemic heart disease	≥75	3204	2.93	1.89	0	1	3	4	9
	Total	6735	6.15	3.16	0	4	6	8	19
	Male	3291	3.01	2.01	0	2	3	4	14
	Female	3444	3.15	2.07	0	2	3	4	12
	65–74	2871	2.62	1.87	0	1	2	4	11
	≥75	3864	3.53	2.16	0	2	3	5	13
Cerebrovascular disease	Total	8202	7.49	3.20	0	5	7	9	21
	Male	4342	3.97	2.14	0	2	4	5	15
	Female	3860	3.53	2.09	0	2	3	5	11
	65–74	3790	3.46	2.02	0	2	3	5	12
	≥75	4412	4.03	2.32	0	2	4	5	14

Abbreviation: SD, standard deviation: Px, xth percentiles; Min., minimum; Max., maximum.

**Table 2:** Summary statistics of daily meteorological factors and air pollution concentrations in Beijing (2009–2011).

Daily Data	Mean	SD	Min.	P_25_	P_50_	P_75_	Max.
Meteorological factors							
DTR (°C)	9.85	3.58	1.10	7.30	9.70	12.10	21.90
In warm season	9.89	3.53	2.10	7.40	9.70	12.00	21.90
In cold season	9.81	3.62	1.10	7.00	9.80	12.10	21.10
Mean temperature (°C)	13.15	11.54	−12.50	1.80	14.90	24.20	34.50
Relative humidity (%)	50.38	19.79	9.00	33.00	51.00	67.00	92.00
Mean wind speed (m/s)	2.23	0.94	0.50	1.50	2.10	2.70	6.40
Atmospheric pressure (hpa)	1012.38	10.25	989.70	1004.10	1011.80	1020.60	1037.30
Air pollutant concentrations							
PM_10_ (μg/m^3^)	110.16	63.28	11.00	58.00	104.00	152.00	544.00
SO_2_ (μg/m^3^)	28.07	28.39	5.00	9.00	18.00	35.00	234.50
NO_2_ (μg/m^3^)	51.88	23.60	11.20	35.20	46.40	62.40	241.60

Abbreviation: SD, standard deviation: P_x_, xth percentiles; Min., minimum; Max., maximum.

**Table 3:** Percent change (mean and 95% CI) of the elderly ER admissions associated with a 1 °C variation in DTR by gender in Beijing during 2009–2011 ^a^.

Gender	Lag	All Cardiovascular Disease	Hypertensive Disease	Ischemic Heart Disease	Cerebrovascular Disease
Male	Single-day lag				
	0	0.36 (−0.23~0.95)	−0.46 (−1.61~0.70)	−0.14 (−1.23~0.96)	0.81 (−0.08~1.70)
	1	0.42 (−0.18~1.03)	0.16 (−1.04~1.38)	0.88 (−0.24~2.01)	0.61 (−0.32~1.54)
	2	0.41 (−0.18~1.00)	0.19 (−0.99~1.39)	0.82 (−0.26~1.91)	0.49 (−0.41~1.40)
	3	0.39 (−0.18~0.96)	0.40 (−0.75~1.57)	0.78 (−0.26~1.84)	0.87 (−0.01~1.76)
	4	0.26 (−0.30~0.82)	0.63 (−0.50~1.78)	0.00 (−1.02~1.03)	0.41 (−0.46~1.29)
	Cumulative-day lag				
	01	0.61 (−0.13~1.36)	−0.25 (−1.68~1.21)	0.57 (−0.81~1.96)	1.08 (−0.02~2.19)
	02	0.86 (−0.01~1.73)	−0.09 (−1.75~1.60)	1.12 (−0.49~2.75)	**1.33 (0.06~2.61)**
	03	**1.04 (0.09~2.00)**	0.19 (−1.64~2.06)	1.56 (−0.20~3.35)	**1.95 (0.44~3.48)**
	04	**1.13 (0.11~2.16)**	0.59 (−1.39~2.62)	1.43 (−0.45~3.34)	**2.13 (0.51~3.77)**
Female	Single-day lag				
	0	**1.01 (0.43~1.59)**	0.83 (−0.15~1.83)	**1.26 (0.22~2.31)**	**1.01 (0.06~1.97)**
	1	**0.90 (0.30~1.50)**	0.23 (−0.85~1.33)	0.79 (−0.28~1.86)	**1.07 (0.09~2.07)**
	2	0.14 (−0.45~0.73)	0.08 (−0.97~1.13)	0.47 (−0.57~1.52)	0.24 (−0.73~1.22)
	3	−0.27 (−0.84~0.30)	−0.33 (−1.34~0.69)	−0.38 (−1.39~0.64)	0.18 (−0.77~1.13)
	4	−0.07 (−0.63~0.49)	−0.22 (−1.21~0.79)	−0.24 (−1.24~0.77)	−0.21 (−1.15~0.73)
	Cumulative-day lag				
	01	**1.46 (0.74~2.19)**	0.68 (−0.67~2.05)	**1.55 (0.26~2.86)**	**1.54 (0.37~2.73)**
	02	**1.46 (0.62~2.31)**	0.66 (−0.91~2.26)	**1.71 (0.21~3.23)**	**1.55 (0.19~2.93)**
	03	**1.16 (0.22~2.10)**	0.35 (−1.37~2.10)	1.30 (−0.34~2.97)	**1.53 (0.03~3.04)**
	04	0.99 (−0.03~2.02)	0.16 (−1.69~2.04)	1.06 (−0.70~2.85)	1.29 (−0.31~2.92)

**^a^** The effects of DTR were estimated by core model. All models controlled for time trend, DOW, holiday, weather conditions and air pollutants. Statistically significant effect estimates were bolded.

**Table 4:** Percent change (mean and 95% CI) of the elderly ER admissions associated with a 1 °C variation in DTR by age group among males and females in Beijing during 2009–2011 ^a,b^.

Group	All Cardiovascular Disease	Hypertensive Disease	Ischemic Heart Disease	Cerebrovascular Disease
Male				
65–74	1.33 (−0.24~2.92)	0.88 (−0.85~2.64)	1.08 (−1.47~3.70)	1.10 (−1.28~3.54)
≥75	**1.31 (0.11~2.52)**	0.65 (−0.85~2.17)	2.17 (−0.04~4.44)	**2.94 (0.90~5.01)**
Female				
65–74	0.74 (−0.29~1.78)	0.22 (−1.15~1.61)	1.84 (−0.22~3.94)	0.22 (−1.42~1.90)
≥75	**2.23 (1.24~3.22) ** *	**1.53 (0.11~2.98)**	**1.85 (0.15~3.57)**	**3.22 (1.30~5.18) ** *

**^a^** The effects of DTR were estimated by core model. All models controlled for time trend, DOW, holiday, weather conditions and air pollutants. Statistically significant effect estimates were bolded; ^b^ The greatest effects of cumulative-day lag (L04, L03 and L04) DTR and single-day lag 4 (L4) DTR were used for all cardiovascular disease, ischemic heart disease, cerebrovascular disease and hypertensive disease ER admissions in males; cumulative-day lag (L01, L02 and L02) DTR and single-day lag 0 (L0) DTR were used for all cardiovascular disease, ischemic heart disease, cerebrovascular disease and hypertensive disease ER admissions in females; * Differences between 65–74 years and ≥75 years in the same gender group, *p* < 0.05.

**Table 5:** Percent change (mean and 95% CI) of the elderly ER admissions associated with a 1°C variation in DTR by season among males and females in Beijing during 2009–2011 ^a,b^.

Season ^c^	All Cardiovascular Disease	Hypertensive Disease	Ischemic Heart Disease	Cerebrovascular Disease
Warm season				
Male	1.21 (−0.11~2.54)	−0.03 (−1.65~1.62)	1.65 (−0.61~3.95)	**3.03 (1.06~5.03) ** *
≥75	**1.81 (0.16~3.84)**	−0.13 (−2.28~2.07)	2.67 (−0.19~5.61)	**4.95 (2.25~7.73) ** *
Female	**1.07 (0.09~2.06)**	−0.13 (−1.62~1.38)	1.15 (−0.80~3.13)	**2.20 (0.43~3.99)**
≥75	**1.76 (0.48~3.06)**	0.87 (−1.11~2.90)	1.10 (−1.13~3.38)	**3.98 (1.33~6.70)**
Cold season				
Male	1.07 (−0.38~2.55)	1.14 (−0.42~2.72)	1.67 (−0.79~4.19)	0.07 (−2.07~2.27)
≥75	0.98 (−0.89~2.87)	1.24 (−0.82~3.34)	1.85 (−1.26~5.05)	0.36 (−2.63~3.43)
Female	**1.93 (0.86~3.01)**	1.28 (−0.16~2.75)	**2.42 (0.23~4.65)**	0.42 (−1.59~2.47)
≥75	**2.63 (1.16~4.11)**	**2.16 (0.13~4.23)**	**2.86 (0.30~5.49)**	2.55 (−0.40~5.59)

**^a^** The effects of DTR were estimated by core model. All models controlled for time trend, DOW, holiday, weather conditions and air pollutants. Statistically significant effect estimates were bolded; ^b^ The greatest effects of cumulative-day lag (L04, L03 and L04) DTR and single-day lag 4 (L4) DTR were used for all cardiovascular disease, ischemic heart disease, cerebrovascular disease and hypertensive disease ER admissions in males; cumulative-day lag (L01, L02 and L02) DTR and single-day lag 0(L0) DTR were used for all cardiovascular disease, ischemic heart disease, cerebrovascular disease and hypertensive disease ER admissions in females; ^c^ Product term of a DTR and season (binary variables for cold season and warm season) was added to the core model; * Differences between warm season and cold season in the same gender and age group, *p* < 0.05.

**Table 6:** Percent increase (mean and 95% CI) of the elderly ER admissions for 1 °C increase of DTR before and after adjustment for covariates ^a,b^.

Covariates	All Cardiovascular Disease	Hypertensive Disease	Ischemic Heart Disease	Cerebrovascular Disease
Male				
None	**1.58 (0.67~2.50)**	1.05 (−0.07~2.18)	1.46 (−0.08~3.02)	**2.22 (0.76~3.70)**
T	**1.18 (0.20~2.17)**	0.57 (−0.56~1.71)	1.18 (−0.49~2.89)	**2.12 (0.59~3.68)**
T + RH + W + P	**1.18 (0.20~2.18)**	0.65 (−0.48~1.80)	1.25 (−0.42~2.95)	**2.09 (0.55~3.66)**
T + RH + W + P + Z	**1.13 (0.11~2.16)**	0.63 (−0.50~1.78)	1.56 (−0.20~3.35)	**2.13 (0.51~3.77)**
Female				
None	**1.53 (0.85~2.21)**	0.49 (−0.49~1.49)	**1.55 (0.18~2.94)**	**2.29 (1.06~3.54)**
T	**1.60 (0.91~2.30)**	0.65 (−0.39~1.69)	**1.69 (0.24~3.16)**	**2.14 (0.86~3.43)**
T + RH + W + P	**1.53 (0.83~2.24)**	0.68 (−0.28~1.66)	**1.60 (0.17~3.05)**	**2.06 (0.77~3.36)**
T + RH + W + P + Z	**1.46 (0.74~2.19)**	0.83 (−0.15~1.83)	**1.71 (0.21~3.23)**	**1.55 (0.19~2.93)**

**^a^** The effects of DTR were estimated by GAM model. All models controlled for time trend, DOW, holiday. Statistically significant effect estimates were bolded; ^b^ The greatest effects of cumulative-day lag (L04, L03 and L04) DTR and single-day lag 4 (L4) DTR were used for all cardiovascular disease, ischemic heart disease, cerebrovascular disease and hypertensive disease ER admissions in males; cumulative-day lag (L01, L02 and L02) DTR and single-day lag 0 (L0) DTR were used for all cardiovascular disease, ischemic heart disease, cerebrovascular disease and hypertensive disease ER admissions in females. Abbreviations: T, mean temperature, RH, relative humidity, W, mean wind speed, P, atmospheric pressure, Z, air pollutants (PM_10_, NO_2_ and SO_2_).

## Abbreviations

ER	Emergency Room
ICD-10	International Classification of Disease, tenth revision
DTR	Diurnal Temperature Range
GAM	Generalized Additive Model
PACF	Partial Autocorrelation Function
T	Mean Temperature
RH	Relative Humidity
W	Mean Wind Speed
P	Atmospheric Pressure
SD	Standard Deviation

## Appendix

**Table A1:** Percent increase (mean and 95% CI) of the elderly ER admissions with a 1 °C variation in mean temperature and a 10-μg/m^3^ increase in air pollutant concentrations by gender in Beijing during 2009–2011 ^a^.

Covariates	All Cardiovascular Disease	Hypertensive Disease	Ischemic Heart Disease	Cerebrovascular Disease
Male				
Temperature ^b^	**0.89 (0.05~1.73)**	−0.21 (1.06~0.65)	**1.62 (0.03~3.23)**	−0.13 (−0.78~0.52)
PM_10 _^c^	0.08 (−0.27~0.44)	0.16 (−0.52~0.84)	−0.56 (−1.23~0.11)	−0.09 (−0.62~0.44)
NO_2 _^c^	0.55 (−0.58~1.68)	0.37 (−1.73~2.52)	−1.16 (−3.22~0.95)	0.42 (−1.21~2.08)
SO_2 _^c^	0.19 (−0.91~1.30)	0.21 (−1.78~2.24)	−0.70 (−2.70~1.34)	−0.22 (−1.84~1.42)
Female				
Temperature ^b^	0.49 (−0.31~1.30)	−0.58 (−1.30~0.14)	0.37 (−0.61~1.36)	0.40 (−0.29~1.09)
PM_10_^c^	**0.45 (0.11~0.79)**	0.44 (−0.14~1.03)	0.01 (−0.60~0.63)	0.52 (−0.03~1.08)
NO_2_^c^	**1.68 (0.62~2.74)**	0.33 (−1.49~2.19)	0.52 (−1.37~2.45)	**2.80 (1.10~4.53)**
SO_2_^c^	0.64 (−0.42~1.72)	−0.55 (−2.31~1.23)	−0.05 (−1.92~1.85)	**2.17 (0.50~3.86)**

**^a^** The effects were estimated by GAM model. We used current day mean temperature, humidity, wind, pressure and pollutant concentrations (lag 0). Statistically significant effect estimates were bolded; ^b^ All models controlled for the time trend, DOW, holiday, humidity, wind, pressure and air pollutants; ^c^ All models were controlled for the time trend, DOW, holiday, mean temperature, humidity, wind, pressure.
